# Phylogenomic and synteny analysis of BAHD and SCP/SCPL gene families reveal their evolutionary histories in plant specialized metabolism

**DOI:** 10.1098/rstb.2023.0349

**Published:** 2024-09-30

**Authors:** Thomas Naake, John C. D'Auria, Alisdair R. Fernie, Federico Scossa

**Affiliations:** ^1^ European Molecular Biology Laboratory (EMBL), Heidelberg, Germany; ^2^ European Molecular Biology Laboratory (EMBL), Hamburg, Germany; ^3^ Leibniz Institute of Crop Plant Genetics and Crop Plant Research (IPK) OT Gatersleben, Seeland, Germany; ^4^ Max Planck Institute of Molecular Plant Physiology, Potsdam-Golm, Germany; ^5^ Council for Agricultural Research and Economics, Research Center for Genomics and Bioinformatics, Rome, Italy

**Keywords:** BAHD, SCPL, acyltransferase, phylogenomics, synteny, secondary metabolism

## Abstract

Plant chemical diversity is largely owing to a number of enzymes which catalyse reactions involved in the assembly, and in the subsequent chemical modifications, of the core structures of major classes of plant specialized metabolites. One such reaction is acylation. With this in mind, to study the deep evolutionary history of BAHD and the serine-carboxypeptidase-like (SCPL) acyltransferase genes, we assembled phylogenomic synteny networks based on a large-scale inference analysis of orthologues across whole-genome sequences of 126 species spanning Stramenopiles and Archaeplastida, including *Arabidopsis thaliana*, tomato (*Solanum lycopersicum*) and maize (*Zea mays*). As such, this study combined the study of genomic location with changes in gene sequences. Our analyses revealed that serine-carboxypeptidase (SCP)/serine-carboxypeptidase-like (SCPL) genes had a deeper evolutionary origin than BAHD genes, which expanded massively on the transition to land and with the development of the vascular system. The two gene families additionally display quite distinct patterns of copy number variation across phylogenies as well as differences in cross-phylogenetic syntenic network components. In unlocking the above observations, our analyses demonstrate the possibilities afforded by modern phylogenomic (syntenic) networks, but also highlight their current limitations, as demonstrated by the inability of phylogenetic methods to separate authentic SCPL acyltransferases from standard SCP peptide hydrolases.

This article is part of the theme issue ‘The evolution of plant metabolism’.

## 1. Introduction

The shared ancestry of genes—especially for members of large gene families—cannot be safely inferred without considering the physical location of genes across genomes. Hence, determining if two orthologues are syntenic or not, that is, if they share the same position and genomic context, is an important reinforcement of the concepts of orthology and homology [1]. The concept of synteny was initially introduced to simply define genes lying on the same chromosome [2], but in its current usage, it now encompasses the conservation of gene order and content in intraspecies and interspecies genome comparisons [3]. As such, synteny, in its more recent definition, is the hallmark of comparative genomic studies, and with the availability of genome information from various organisms, it has been detected across many evolutionary lineages to establish chromosome rearrangements and ancient polyploidization events [4–6].

The conservation, or, on the other hand, the rupture of gene order, are important factors reflecting possible functional implications. If two orthologous genes share the same genomic context, i.e. if they are topoorthologues [1], they may be subjected to similar patterns of transcriptional activity [7]. In some types of gene duplication modalities (especially in segmental duplication or retroduplication), the genomic context is not necessarily conserved [8,9], a situation which may favour functional diversification and the emergence of novel phenotypes. The structural and functional differences of cell walls of Eudicots with respect to those of Poaceae, for example, have been traced back to markedly different genomic landscapes—in terms of synteny relationships and copy number variation (CNV)—of the main gene families involved in cell wall biosynthesis [10].

If a conserved genomic context may constrain gene function and its regulation, then the dispersion of the conserved gene order (synteny rupture) relaxes the constraint and allows for accelerated neofunctionalization. Such dispersals are common and may result as a consequence of various types of genetic events, e.g. genomic/chromosomal rearrangements associated with gene loss, transposition or chromosome rearrangements. These may modify the gene(s) expression pattern and eventually lead to the emergence of phenotypic novelty. This recurrent evolutionary principle, i.e. mutation, sequence and/or regulatory divergence and selection, has been well investigated with regard to morphological innovations, especially in the animal kingdom [11], but, with very few notable exceptions [12,13], less attention has been dedicated to the study of metabolic innovations in the plant kingdom. The chemical diversity of plant metabolism, amounting to several hundred thousands of distinct chemical entities, reflects the adaptations to the disparity of ecological niches plants occupy today. Since the colonization of land around 500 Ma, early terrestrial plants have needed to survive in new environments where new types of chemistry were needed, e.g. the phenylpropanoid backbone to reflect higher light intensities or the rigid polymer lignin to sustain vertical growth [14,15]. These metabolic innovations emerged following various events of ancient genome duplications, a much more common motif in the evolution of plants with respect to all other kingdoms of life, which generated the raw genetic material upon which selection could act [16].

Today, plants’ chemical diversity can be attributed to a relatively restricted number of distinct enzyme families, each one often including hundreds of genes within a single genome. In inferring shared ancestry, phylogenetic analysis based on multisequence alignments may often lead to ambiguous results, owing to the masking effects that genome duplications and complex chromosome rearrangements may have in establishing clear phylogenetic relationships [17]; phylogenomic approaches, including synteny analysis, are instead highly informative of the evolutionary events shaping the size of gene families and in defining the extent of positional conservation, as they may uncover chromosome rearrangements and the shared ancestry of the members of a gene family [18]. For example, the integration of phylogenetic and comparative synteny analyses between maize (*Zea mays*) and sorghum (*Sorghum bicolor*) was essential to detect the ancestral chromosome condensations of the sorghum genome, explaning why single arms of different maize chromosomes are syntenic to full sorghum chromosomes [4,19–21].

The enzymes encoded by the main metabolic gene families in plant genomes usually synthesize a limited variety of ‘core’ structures, which are then modified in a combinatorial fashion by the addition of several ‘decorating’ groups. Acyltransferases (EC: 2.3.-.-) may be involved both in the synthesis of the core chemical structures and in their subsequent modifications. One of the most common types of modifications is the addition of an acyl group to form esters and amides. In plants, acylated compounds are largely the products of only two distinct gene families, the BAHD [22] and the serine-carboxy peptidase-like (SCPL) acyltransferases [23,24]. At least in Angiosperms, BAHD acyltransferases catalyse the acylation of many classes of specialized metabolites (phenylpropanoids, various types of alkaloids, terpenoids, epicuticular waxes), using a CoA thioester as the activated acyl donor. Esters are the predominant outcome, but occasionally they also generate amides via *N*-acylation. BAHD acyltransferases constitute a medium-sized gene family: Algae contain one or a few copies, but the BAHD copy number increased markedly upon the colonization of land, perhaps the evolutionary transition with the greatest impact on plants’ metabolic capability [25,26]. Arabidopsis, Medicago and Vitis each contain around 50 BAHD genes, but some species harbour over 140 BAHD genes [27]. BAHD genes are divided into at least eight major clades by phylogenetic analyses, but being in the same clade (i.e. sharing a strong sequence similarity) is not always a sign of having similar substrate specificity or enzymatic activity, which makes it extremely challenging to infer evolutionary relationships based solely on phylogenetics [27].

SCPL acyltransferases rather use glucose esters, as the activated acyl donors, and acylate a wide variety of substrates including carbohydrates, ammonium compounds, organic acids, phenylpropanoids and auxin conjugates. Sinapate esters, for example, are abundant in Brassicales [28], but in general, glucose esters have been reported from several other orders/families of Spermatophytes (see the electronic supplementary material, table S6, for a survey of the occurrences of the main glucose esters in other plant families). SCPL acyltransferases derive from serine carboxypeptidases (SCPs), which act as proteases cleaving the peptide bond at the carboxy terminus of a polypeptide chain. Intriguingly, SCPL acyltransferases have conserved the catalytic triad (Ser-Asp-His) typical of carboxypeptidases, but have lost the protease activity. In contrast to the BAHD family, less attention has been afforded to studies of the phylogeny of SCP/SCPL members. The Chlorophyta contains only a few SCP/SCPL sequences. However, the size of the family increases with the move to terrestrial habitats (51 SCP/SCPL genes in Arabidopsis, over 100 in *Brassica napus*) [29,30].

The existence of two distinct gene families whose members can catalyse acylation reactions starting from different acyl donors, but leading, for example in the case of phenolics, to the same set of products [24], led us to investigate whether the same gene family dynamics (e.g. CNV, potential conservation of synteny and phylogenetic diversity) underlies this apparent convergence of metabolic activity. To do so, we first had a look at the distribution of BAHD and SCP/SCPL genes across a selection of high-quality genomes of 126 photosynthetic organisms, from Cyanobacteria to Angiosperms, to then analyse the synteny relationships of these two gene families across this set of genomes. Phylogenomic analyses, combined with the investigation of the structure of synteny networks, highlighted different patterns of distribution and diversification of the members of these two gene families, with varying extents of synteny conservation.

## 2. Results and discussion

### (a) Emergence and initial expansion of BAHD and serine carboxypeptidase/serine carboxypeptidase-like genes

**Figure 1.**
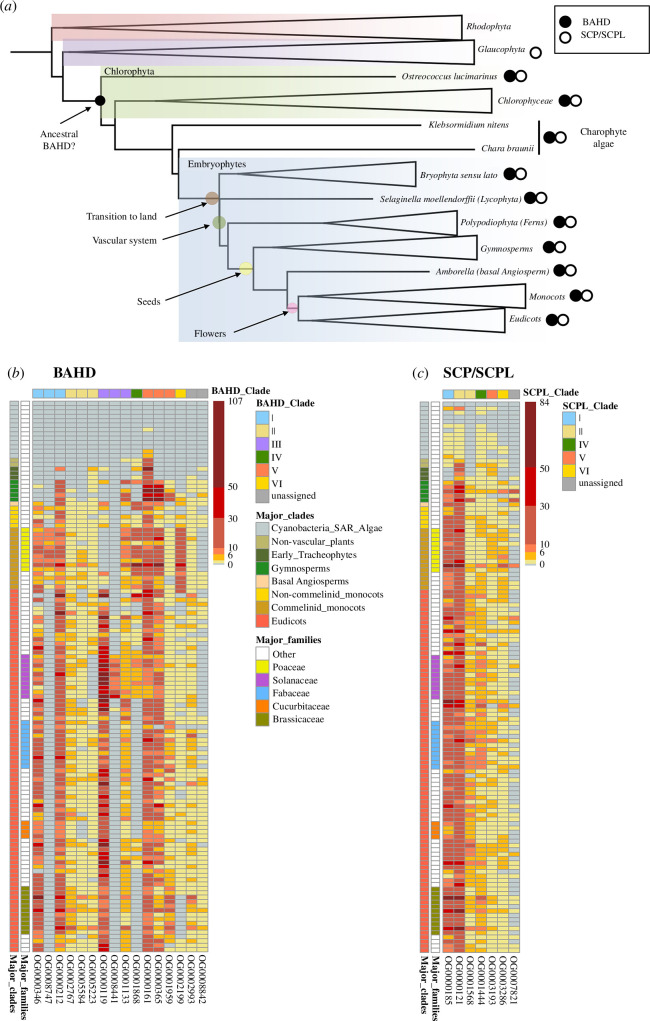
Species distribution of BAHD and SCP/SCPL genes. (*a*) Simplified phylogenetic tree of the Archaeplastida showing the presence of BAHD and SCP/SCPL genes across the main taxonomic groups. The internal node in which the ancestral BAHD gene could have emerged is indicated by the arrow and has been identified by parsimony considering the presence/absence of BAHD (black dots) in terminal nodes. SCP genes have a deeper evolutionary origin, given that they are present also in fungi, animals, [33]) and, in our species set, they have been detected in Stramenopiles (not shown in the phylogenetic tree). The main evolutionary innovations in the green lineage are marked by coloured circles. (*b*) and (*c*) Heatmaps representing copy number of BAHD (*b*) and SCP/SCPL genes (*c*) across the 126 genomes analysed (represented on the rows, with the major organism groups/taxonomic clades and Angiosperm families highlighted with specific colours). BAHD and SCP/SCPL orthogroups, as defined by OrthoFinder, are represented on the columns, and have been assigned to the traditional classification in different clades on the basis of the published phylogenetic analyses for BAHD and SCPL acyltransferases. In particular, for BAHD genes, clade numbering is based on [27,34,35], with the addition of clade VI (OG0002199), which includes, among others, the BAHD genes involved in the synthesis and feruloylation of arabinoxylan [36]. Clade numbering for SCP/SCPL genes revises the classification reported in [29] and [37], with clade I (OG0000185) now including the SCP/SCPL genes formerly attributed to clades Ia and Ib. Cell colour indicates the number of detected genes, ranging from 0 (grey, no BAHD or SCP/SCPL genes detected) to the maximum number of genes detected in each species/orthogroup (107 for BAHD and 84 for SCP/SCPL, in dark red). Orthogroups containing less than 100 genes are not shown.

To quantify the copy number of BAHD and SCP/SCPL genes across the genomes of the 126 species analysed here, we queried the data obtained from OrthoFinder v. 2.4.0 [31] and Markov clustering (MCL, [32]), which, starting from the full proteome data of each species, provided a classification of all encoded proteins into orthogroups (OGs) and MCL groups. OGs are defined as group of genes descending from a unique single gene in the last common ancestor of a group of species, and we will adopt this definition here as a synonym for protein (sub)family. Our 126 species set included one member of the Cyanobacteria (Synechocystis), two Stramenopiles (syn. Heterokontophyta) and 123 Archaeplastida (Plantae *sensu latu*), including 10 Algae (belonging to Rhodophyta, Glaucophyta, Chlorophyta and Charophyta) and 113 Streptophyta, ranging from *Marchantia polymorpha* (as a representative of a non-vascular plant) to more than 100 genomes of Tracheophytes (Lycophyta, Polypodiophyta, Gymnosperms and Angiosperms) (figure 1*a*; electronic supplementary material, table S1, for a full list of the species under investigation). In the following discussion, although we will present data about the emergence of subclades of BAHD and SCP/SCPL in relation to the specific taxonomic groups where they were first identified, this is intended to be limited to the set of species under examination, and, as such, is not meant to exclude the possible presence of BAHD or SCP/SCPL genes in species not included in the abovementioned species set.

From the gene count data of all species, we extracted the OGs related to the BAHD and SCP/SCPL families (table 1; electronic supplementary material, table S2), and plotted their copy numbers in a heatmap (figure 1*b,c*). The first BAHD gene to be detected emerges—perhaps episodically—in Ostreococcus (Chlorophyta), but several BAHD genes, up to five copies, are consistently detected in Charophyte algae, *Klebsormidium nitens* and *Chara braunii*, which are still mostly freshwater organisms but may also occur in moist environments and brackish waters. These early BAHD genes belong to OG0000161, part of a large clade of BAHD genes (clade V) which includes, among others, also those members producing hydroxycinnamoyl shikimate/quinate esters that are used in lignin biosynthesis in land plants. One of the genes found in *C. braunii* was indeed shown to act *in vitro* as a hydroxycinnamoyl-CoA:quinatehydroxycinnamoyl transferase (CbHQT, [27]), and the ability to form quinate esters could thus be considered the ancestral BAHD activity.

**Table 1.** List of orthogroups for BAHD and SCP/SCPL genes.

BAHD genes^a^					
orthogroup	orthoMCL group	clade	number of genes	bait genes	phylogenetic distribution
OG0000346	group_414	I	1334	NtMAT1 (BAD9 256g0050.1, [38])	Angiosperms
OG0008747	group_414	I	106		strictly monocot-specific OG
OG0040891	group_414	I	3		members only from *O. rufipogon* and *O. sativa* (wild and domesticated rice)
OG0000212	group_298	I	1711	AT5G67160 (AtEPS1, enhanced Pseudomonas susceptibility1, SA biosynthesis, [39]), AT5G23940 (DCR, defective in cuticular ridges, [40])	Spermatophytes (seed plants)
OG0002767	group_1084	II	305	AT4G24510.1 (CER2, [41])	Spermatophytes (seed plants)
OG0005584	group_1084	II	178		Angiosperms
OG0005223	group_1084	II	185		Tracheophytes (vascular plants)
OG0000119	group_179	III	2426	Capang02g002092 (AT3-D1, CS [42]), also tomato acylsugar-ATs: SlASAT1 (Solyc12g006330), SlASAT2 (Solyc04g012020), SlASAT3 (Solyc11g067270) and SlASAT4 (Solyc01g105580) [43–45]	Tracheophytes (vascular plants), but members are absent in Poaceae. Tomato acylsugar-related ATs cluster here
OG0008441	group_179	III	122	SlSpmHT (Solyc12g010980, [46])	mostly Solanaceae-specific OG
OG0014841	group_179	III	14		Rutaceae-specific OG
OG0061075	group_179	III	2		Petunia-specific OG
OG0028240	group_179	III	4		
OG0001133	group_56	III	573	peaxi162Scf00474g00217.1 (homologous to PhCFAT, ABG75942)	Spermatophytes (seed plants)
OG0001868	group_56	IV	402	Os09g37200 (a feruloyl putrescine N-transferase, [47]), Os04g56910 (coumaroyl agmatine transferase, [47]), Os09g37180 (putative putrescine *N*-acyltransferase, [48]), Solyc11g071470 (SlPHT, putrescine-hydroxycinnamoyltransferase), Solyc11g071480 (another putrescine-HT)	Embryophytes (land plants), but absent from most Superrosids. BAHD involved in amine acylation (synthesis of hydroxycinnamic acid amides, HCAAs) cluster here
OG0000161	group_56	V	2023	AtHCT (NP_199704.1, AT5G48930.1, clade Vb), AtASFT (aliphatic suberin feruloyl transferase, Q94CD1, At5g41040, clade Va), AtSHT (At2g19070, spermidine hydroxycinnamoyl transferase, belongs to clade Vb), SlHQT (CAE46933, Solyc07g005760, clade Vb), NtHQT (CAE46932, Nitab4.5_0002320g0020.1, clade Vb), plus Os06g08580, Os06g08610, and Os06g08640, a cluster controlling coumaroyl putrescine levels [48], SlASFT (Solyc03g097500, aliphatic suberin feruloyl transferase (ASFT), [49]), Solyc07g015960 (SlSHT, spermidine hydroxycinnamoyl transferase)	Chlorophyta (*Ostreococcus lucimarinus*), Stramenopiles and Embryophytes; some amine ATs also here
OG0000365	group_56	V	1307	AtCHAT (AAN09797, AT3G03480, clade Va), CmAAT2 (AAL77060, MELO3C024766P2), NtBEBT (Nitab4.5_0000130g0220.1, AAN09798, belongs to clade Va), Peaxi162Scf00007g00011.1 (homologous to PhBPBT, AAU06226, clade Va)	Spermatophytes (seed plants)
OG0001959	group_56	V	390	AtSDT (NP_179932, At2g23510, [50]), AtSCT (At2g25150 [50]), both in clade Va	Spermatophytes (seed plants)
OG0002199	group_56	VI	358	At3g62160 [36], OsFMT (feruloyl-CoA monolignol transferase, Os05g19910), OsPMT (*p*-coumaroyl CoA monolignol transferase, Os01g18744) these two genes in rice are monolignol transferases [51], also Zm00001d035246 (ZmFMT, feruloyl-CoA monolignol transferase)	Spermatophytes (seed plants), with a large increase in copy number in Monocots. Includes all known ATs acylating AX/GAX and monolignol transferases. No members from Brassicaceae.
OG0002993	group_56	unassigned	286		Spermatophytes (seed plants)
OG0008842	group_56	unassigned	100		Embryophytes (land plants), but no members from Poaceae and several species of both Asterids and Rosids
OG0008898	group_56	unassigned	95		
OG0028346	group_56	unassigned	5		members only from *O. rufipogon* and *O. sativa* (wild and domesticated rice)
OG0025243	group_56	unassigned	6		*Selaginella*-specific OG

**SCP/SCPL genes** ^b^					
orthogroup	orthoMCL group	clade	number of genes	bait genes	phylogenetic distribution
OG0000185^c^	group_50	I	1885	Arabidopsis: At2g22920 (AtFPT1, AtSCPL12, flavonol-phenylacyltransferase 1, [52]), At2g22960 (AtFPT2, flavonol-phenylacyltransferase 2, [52]), At2g22990 (SMT, 1-O-sinapoyl-b-glucose:L-malate sinapoyltransferase, AAF78760, SNG1, [53]), At2g23000 (SAT, 1-O-sinapoyl-b-glucose:anthocyaninsinapoyltransferase, AEC07395 [54]), At2g23010 (SST, 1-O-sinapoyl-β-glucose:1-O-sinapoyl-β-glucose sinapoyltransferase, [54]), At3g12203(SCPL17, a benzoyl/sinapoyltransferase acting on glucosinolatesAAS99709, [55]), At5g09640 (*sng2*, 1-O-sinapoyl-b-glucose:cholinesinapoyltransferase, AAK52316, [56]). This OG includes all Arabidopsis genes (from 1 to 21 according to [29]). *B. napus*: BnA02g0049730 (*BnSCT1,* GSBRNA2T00143572001), BnA02g0049710 (*BnSCT2*) [57] *C. sinensis*: TEA034032.1 (CsSCPL4, EC-GT, galloylglucose:epicatechin acyltransferase, UPO25246, [58]), TEA023444.1 (CsSCPL5, the non-catalytic paralog of CsSCPL4, UPO25247, [58]) *D. carota*: DCAR_029167 (DcSAT1/DcSCPL1, sinapoylglucose:anthocyaninsinapoyltransferase 1, QEA09237 [59,60] *O. sativa*: Os02g26480 (indoleacetylglucose:inositol acyltransferase, IAA-AT, EEE56946.1, [61]) *S. pennellii*: Sopen10g020280.1 (isobutyrylglucose:isobutyrylglucose acyltransferase, GAT, Q9LKY6 [62,63]) *V. vinifera*: GSVIVT01037059001 (*VvGAT1*, glucose acyltransferase 1, AVK87731), GSVIVT01037791001 (*VvGAT2*, glucose acyltransferase 2, AVK87732) [64] *Z. mays*: Zm00001d016459 (NP_001132320, 1-O-(indole−3-acetyl)-β-D-glucose: myo-inositol indoleacetyl transferase, IAInos synthase, [65])	Diaphoretickes (Stramenopiles and Archaeplastida)
OG0000121	group_50	II	2397	includes the genes of Clade II from 22 to 44 [29], At4g30610 (brassinosteroid suppressor 1, BRS1, [66])	Diaphoretickes (Stramenopiles and Archaeplastida)
OG0001568	group_50	III	458	includes genes 45 and 46 of Clade II from [29]	OG emerges in Ferns; members are distributed across all Spermatophytes (seed plants)
OG0001444	group_50	IV	484	includes genes 47, 48 and 49 from [29]	Diaphoretickes (Stramenopiles and Archaeplastida)
OG0003193	group_50	V	271	includes gene 50 (At1g15000) from [29]	Diaphoretickes (Stramenopiles and Archaeplastida)
OG0003286	group_50	VI	264	includes gene 51 (At2g27920) from [29]	Viridiplantae (green plants, including Chlorophyta)
OG0007821	group_50	unassigned	140		OG emerges in Ferns, with a patchy distribution across Spermatophytes (seed plants)
OG0048281	group_50	unassigned	2		
OG0019389	group_50	unassigned	9		
OG0027394	group_50	unassigned	5		

^a^ BAHD clades have been assigned according to [27,34,35].

^b^ SCP/SCPL clades have been assigned according to [29,37]. Clade III of SCP/SCPL include genes only from human, *C. elegans* and zebrafish, hence they are not present in this analysis.

^c^ All known biochemically-characterized SCPL acyltransferases cluster in this OG.

After their initial emergence with few gene copies (less than five), the BAHD gene family expanded massively in parallel to two transitions: the first, with the passage from water to a terrestrial environment, and then with the development of the vascular system of terrestrial plants (figure 1*b*). The first expansion can be thus recorded in Marchantia and Physcomitrella (land plants, but not yet relying on a highly differentiated vascular system), with 32 and 15 genes, respectively. The majority of these genes always come from the OG0000161/clade V. The second expansion, occurring in parallel to the acquisition of a conducting system, can be observed with the massive increases of BAHD copy numbers in Lycophyta (*Selaginella moellendorffii*) and Polypodiophyta (Ferns). This second transition, in particular, is accompanied by the emergence of BAHD genes belonging to other clades: *Se. moellendorffii* contains genes belonging to clade I (which are involved, for the major part, in the acylation of flavonoids and phenolic glucosides, [22]); also, additional BAHD genes of clade II (wax biosynthesis) can be identified in *Selaginella* and Polypodiophyta (Ferns). The capacity to acylate phenolic compounds and the synthesis of a hydrophobic barrier thus seems to be essential in the water-to-land transition and the subsequent development of a fully differentiated vasculature.

In comparison to those from the BAHD family, SCP/SCPL genes have a deeper evolutionary origin. Several SCP/SCPL gene copies can be already detected in Heterokontophyta, thus before the divergence of Archeaplastida (i.e. Plantae *sensu lato*, figure 1*c*). Ambiguity however remains whether these ancestral genes act as peptide hydrolases (SCPs) or already as genuine AcylTransferases (SCPL-ATs). Although limited sequence variation in a motif composed of five amino acids has been suggested to discriminate between peptidases (SCPs) from *bona fide* acyltransferases (SCPL-ATs) of clade Ia [67], the scarcity of biochemically characterized enzymes, especially from lower organisms, renders activity prediction for SCP/SCPL genes, on the basis of primary sequence alone, unreliable. One of the species from Heterokontophyta, *Aureococcus aneophagefferens*, contains 13 SCP/SCPL genes; of these, three belong to an OG (OG0000185) which also includes all genuine SCPL-ATs characterized so far (table 1). That said, and given that the three genes from *A. aneophagefferens* of OG0000185 show the typical pentapeptide motif of authentic SCPs (peptidases), it is clear that the phylogenetic placement of SCP/SCPL genes in different OGs/clades cannot, in itself, be predictive of their activity as peptidases or acyltransferases. Indeed, our analyses here suggest that a more accurate assessment of this relationship is a critically needed step in the future characterization of this gene superfamily.

Irrespective of their exact functionality, the emergence of SCP/SCPL genes was detected, starting from Heterokontophyta, with few gene copies (less than four) in at least four OGs (OG0000185, OG0000121, OG0001444 and OG0003193, representing clade I, IIA, IV and V of earlier phylogenetic analyses within the family [29,37], with the exception of OG0001568 and OG0007821, which have instead a later emergence and whose members can be detected starting from Polypodiophyta (Ferns) (figure 1*c*). The initial expansion of SCP/SCPL genes, with an increase in copy number, takes place in Bryophyta (>10 genes) and later in Gymnosperms (with over 30 genes), but it is an increase mostly limited to members of OG0000121 (clade IIa). This pattern of expansion for SCP/SCPL genes of OG0000121 is thus concomitant to the increase of BAHD genes of OG0000161, occurring, in both cases, from Charophyte algae to Bryophyta, in parallel to the water-to-land transition (figure 1*a*).

### (b) General patterns of copy number variation across phylogeny

In addition to establishing the time of emergence and initial expansion, some clear trends in CNV emerge when globally assessing how the members are distributed across species and OGs (figure 1*b,c*). First, some BAHD OGs are phylogenetically restricted to specific groups of organisms. The existence of these OGs could be related to the presence of metabolite classes which specifically accumulate in a phylogenetically restricted manner. A Monocot-specific OG (OG00008747), whose members have been not biochemically characterized, is part of clade I, and, as such, its genes could be related to those BAHD genes which mostly acylate flavonoids and various phenolic glucosides [68]. Another OG, which is mostly restricted to Solanaceae (OG0008441), contains a tomato gene characterized as a spermine hydroxycinnamoyl transferase (SlSpmHT, Solyc12g010980, [46]), and is involved in the synthesis of hydroxycinnamoyl-spermine amides, a rare subgroup of hydroxycinnamic acid amides which are mostly restricted to Solanaceae [69–71]. Second, other BAHD OGs, containing members from most of the species, instead show a massive increase of BAHD gene copies only in specific plant families or taxonomic groups. Gymnosperms experienced an amplification of BAHD genes specifically belonging to three OGs (OG0000212, OG0000161 and OG0000365, corresponding to the previously defined BAHD clades I and V), while all Monocots have a higher number of BAHD genes of OG0002199 (on average, >15) with respect to non-Monocot species, which only contain one or two copies. BAHD genes from Monocots in this OG include all known acyl glucoarabinoxylan/arabinoxylan and monolignol transferases, and catalyse esterification reactions on the hemicelluloses and lignin with ferulic acid or with 4-coumarate. These acylations were once considered exclusive of Monocots, but have since been found to be more common in the cell walls and lignin of Monocots with respect to Eudicots [51,72,73]. CNV and different genomic properties in cell wall biosynthesis and modification genes have in fact been identified as the main drivers of the chemical differences in the cell walls of Monocots (especially Poaceae) with respect to Eudicots [10]. Another characteristic OG showing large CNV across species is OG0001868, which contains, among other genes, several of the known BAHD enzymes characterized so far as amine-*N*-acyltransferases (e.g. OsPHT4, Os09g37200, a rice feruloyl putrescine *N*-transferase [47]). This OG emerges with few copies in non-vascular plants, to then experience a massive increase in copy number in Monocots (which are known to host a large diversity of acylated amines, [48,74,75]), but its members are then absent from several families of Rosids (Fabaceae, Cucurbitaceae and Brassicaceae, figure 1*b*). Amine-N-acyltransferases are, however, also found in another OG covering a large range of species (i.e. OG0000161, which includes, e.g. SlSHT, Solyc07g015960, a tomato spermidine hydroxycinnamoyl transferase [46,76] and a tandem repeat of three rice ATs specific for spermidine, Os06g08580, Os06g08610 and Os06g08640 [48]). Thus, the recovery of BAHD genes of different evolutionary origins, but with the same catalytic activity, points to independent pathways of convergent evolution for the capacity to acylate nitrogen, at least in rice and tomato, but possibly also for the other species possessing genes in both OGs.

SCP/SCPL family genes have, on the other hand, a very distinct pattern of CNV in comparison to BAHD family genes. There are no clear taxonomic groups or plant families lacking entirely SCP/SCPL genes: the most numerous OGs span in fact the full range of species since the time of their emergence (figure 1*c*). A large interspecies variation in copy number occurs mainly in only two OGs, OG0000185 and OG0000121 (figure 1*c*), which represent clade I and part of clade II, respectively, of the previous phylogenetic analyses conducted on this family [29,30,37,77]. The copy number in the remaining SCP/SCPL OGs is, on the other hand, extremely robust across phylogeny, rarely exceeding 10 copies, with most species having from one to four copies. This apparent constraint in the number of SCP/SCPL gene copies is even more evident when looking at specific plant families: Poaceae have two genes (per diploid genome) of OG0001568 (part of clade II); similarly, Solanaceae species have a single gene of OG0003286 (clade VI). The different extent of CNV across SCP/SCPL OGs may suggest that along the multiple whole genome duplication (WGD) events occurring along Embryophyta evolution [12,78], only the incipient paralogues belonging to specific OGs (e.g. OG0000185 and OG0000121) were preferentially retained over the paralogues derived from other different OGs. So, the extreme conservation in copy number observed in some OGs may point to a maladaptive role of an increase in their gene dosage derived from gene duplications. Thus, the ancestral copy number increase in these OGs might have been selected against during the incipient phases following gene duplication, resulting in the extreme robustness of the SCP/SCPL copy number we observe today. Although a gene duplication event does not necessarily imply an immediate doubling of its gene product, it generally leads to an increase [79], and, in the case of enzymes, this is oftentimes correlated to an increased activity [80–82]. As such, this situation is reminiscent of the adaptive hypothesis of gene duplication, where the adaptive value of a gene is undeniably influenced by the dosage of its product or its activity [79,82,83].

### (c) Syntenic communities of BAHD and serine carboxypeptidase/serine carboxypeptidase-like genes across phylogeny

To further investigate the genomic properties of BAHD and SCP/SCPL genes and assess whether the members of these two gene families share a similar extent of positional conservation, we next assembled synteny networks from the genomic coordinates of the 126 species, using the algorithms iADHoRe and MCScanX. A synteny network is an object composed of nodes (representing genomic regions containing a single BAHD or SCP/SCPL gene, or a tandem array of multiple BAHD/SCP copies, from any of the 126 species) and edges, representing a synteny relationship. Each edge has a weight, ranging from 0.25 to 1, assigned whether the link has been detected in all or only some of the synteny algorithms used here (i.e. a weight of 1 corresponds to synteny being detected by both iADHoRe and MCScanX, and on both orthologue datasets produced by OrthoFinder or MCL, see also §4 for details). Detection of these syntenic relationships was conducted within each MCL and OG group individually, given that clustering of orthologues by MCL and OG reflected well, at least for the BAHD family, the traditional classification into different clades. Indeed, four distinct MCL groups identified clades I, II and III, assigning all remaining genes to a single additional MCL group (table 2; electronic supplementary material, table S4 for the full community membership data of the synteny network). This classification is consistent with the phylogenetics of BAHD genes, in which the remaining clades, and especially clade V, are loosely defined [27,84]. In the case of SCP/SCPL genes, MCL clustering assigned all genes to a single group (table 2). In both cases, adopting MCL groups for initial syntenic block detection allowed us to avoid the extreme fragmentation that synteny algorithms would have faced when using individual OGs as the starting datasets. Starting from MCL and OG groups, we thus detected six individual components in the synteny network, five corresponding to BAHD and the remaining one to SCP/SCPL genes (a component is defined as a group of genes/gene regions, from multiple species, which are connected by synteny relationships. Under this definition, it is important to note that components need not be fully connected networks, they could for example be represented by nodes with a single edge). The full network contained 9503 nodes (genes or gene regions); of these, 6002 were part of the five BAHD components, with the remaining 3501 assigned to the single SCP/SCPL component (table 2; electronic supplementary material, table S4).

**Table 2.** Synteny network components and cliques (complete synteny graphs) for BAHD and SCP/SCPL genes.

BAHD genes				
MCL (synteny network component)	clade	orthogroup	number of nodes (genes/regions in the synteny network component)	number of cliques (>2 genes/regions)
group 414	I	OG0000346, OG0008747	567	99
group 298	I	OG0000212	905	138
group 1084	II	OG0002767, OG0005584, OG0005223	499	54
group 179	III	OG0000119, OG0008441	1178	251
group 56	III, IV, V, VI plus unassigned genes	OG0001133, OG0001868, OG0000161, OG0000365, OG0001959, OG0002199, OG0002993, OG0008842	2853	477

SCP/SCPL genes				
MCL (synteny network component)	clade	orthogroup	number of nodes (genes/regions in the synteny network component)	number of cliques (>2 genes/regions)
group 50	I, II, IV, V, VI plus unassigned genes	OG0000185, OG0000121, OG0001568, OG0001444, OG0003193, OG0003286, OG0007821	3501	407

Given that the representation of multispecies synteny relationships from entire components was still highly complex, we focussed on extracting fully connected subnetworks (i.e. cliques) with the aim to represent and analyse the species range of their composing elements. In translating a network construct to the synteny field, we can think of a ‘clique’ as a group of homologous genes (or gene regions)—given that cliques are obtained initially from the MCL and OG clusters of orthologues—which are all fully connected to each other (a clique is thus a complete graph, where synteny relationships are completely transitive). Although the species range in the nodes of a clique underestimates the extent of positional conservation, detecting cliques, and studying their composition, offers a view on the ‘core’ syntenic communities of the network, those where positional conservation is strong enough to be fully reciprocal among the nodes. Homologous genes composing a clique can thus be considered as ‘reinforced’ orthologues, subjected to a greater evolutionary constraint that also encompasses a full reciprocal degree of conservation of their genomic position across a number of species [1]. Changes in genomic context are in fact often related to trait variation, and different genomic contexts are, likewise, associated with different scenarios of transcriptional regulation [85,86]. Thus, the members of a clique, which all retained the genomic organization of their immediate ancestors, may also share coordinated expression and functions of the ancestral gene from which they derive [1,85].

**Figure 2.**
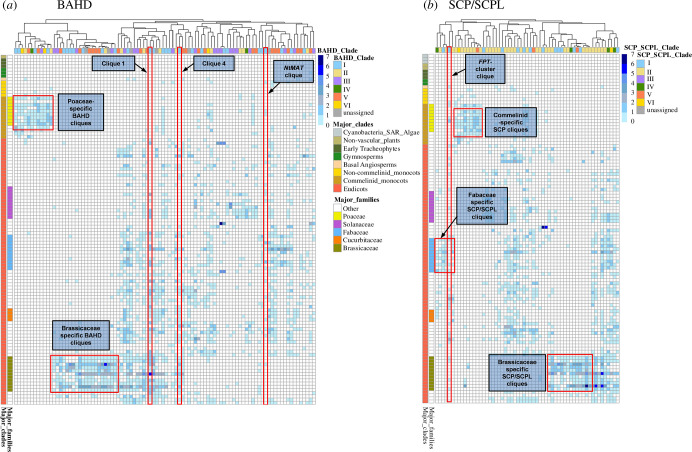
Heatmaps representing the composition of the cliques for BAHD (*a*) and SCP/SCPL genes (*b*) across the species (genomes) making part of the phylosynteny network from which cliques could be extracted. Species are represented on the rows, with the major organism groups/taxonomic clades and Angiosperm families highlighted with specific colours. Cliques (defined as complete synteny subnetworks in which every pair of distinct genes/regions is connected by a unique edge representing a synteny relationship) are represented individually on the columns, with specific colours corresponding to the phylogenetic BAHD or SCP/SCPL clade to which the clique belongs. Columns are clustered according to Pearson’s correlation distance (complete linkage). Only cliques containing members from at least 10 species are represented. Cell colour indicates the number of single genes or gene regions for each species, ranging from 0 (white, no BAHD or SCP/SCPL genes/regions in the clique) to the maximum number of genes/regions detected in each species/clique (seven for both BAHD and SCP/SCPL, in dark blue). Red rectangles indicate cliques or specific areas of the heatmaps discussed in the text.

From the individual network components, we thus extracted the cliques for BAHD and SCP/SCPL and represented their node composition across the set of species (figure 2).

BAHD genes were clustered in 94 cliques (counting only those having, in total, at least 10 genes or gene regions, figure 2*a*); of these, within the top-five cliques with the highest number of genes, three belong to MCL group 298, which contains BAHD genes part of clade 1 (OG0000212, see table 2), and in particular those classified in subclade 1c according to [27]. The few characterized genes in this subclade are *AtDCR* (AT5G23940, DCR, a cutin diacylglycerol AT, [40]) and *AtEPS1* (AT5G67160, enhanced pseudomonas susceptibility 1, an AT involved in salicylic acid metabolism, [87]). These three large cliques contain between 60 and 80 genes/gene regions, and span around 50 species. Although the emergence of members from this OG are first recorded in *Se. moellendorffii* to then expand massively in Gymnosperms (figure 1*a*), establishment of a strong positional conservation can only be detected from a more limited set of species. For clique 1, the largest BAHD clique in terms of number of genes (80) and species (48), and which includes *AtEPS1,* synteny extends from basal Eudicots (Ranunculales) and includes species from Solanales, Vitales, Fabales, Rosales and most of all remaining Rosids in our set. Clique 4, which includes *AtDCR*, comprises 45 genes/gene regions and extends over 40 species (spanning several orders of Asterids and Rosids). In addition to these large cliques, several other BAHD genes are clustered in cliques which have a more limited species range, extending only across specific plant families (figure 2*a*). For example, at least 12 cliques extend only across Poaceae, and include, as would be perhaps expected, a clique composed of clade VI BAHD genes (clade VI BAHD acylate components of type-II cell walls, which are typical of Commelinid monocots [36,72]). Another clique, extending across most of Commelinid monocots, is the one including the three tandem rice genes Os06g08580, Os06g08610 and Os06g08640 (clade V), a locus experimentally shown to control the levels of coumaroyl putrescine [48]. This arrangement of clustered genes is positionally conserved, with a copy number ranging from two to four copies, also in *Leersia perrieri*, *Oryza rufipogon*, *Setaria italica*, *So. bicolor*, *Z. mays*, *Ananas comosus* and *Musa acuminata*, showing that this complex locus, containing the BAHD genes responsible for the synthesis of this aromatic phenolamide, is syntenic across Commelinids.

Cliques extracted from the SCP/SCPL network offer similar cases of positional conservation extended only over single plant families or taxonomic groups (figure 2*b*). As for BAHD, several SCP/SCPL cliques are exclusive to Commelinid monocots. With respect to BAHD, where these Commelinid-specific cliques have different evolutionary origins (being composed by genes from all BAHD clades), in the SCP/SCPL family these cliques are instead almost entirely composed of genes derived from clade II (OG0000121). There are no genuine ATs characterized so far from this clade, and the only known rice gene *(Os05g06660*, *GS5, OsSCP26*) was shown to be a positive regulator of grain size [88,89]. Given that, except from clade I genes, where a single aminoacid change in a pentapeptide around the catalytic Ser discriminated between SCPL-ATs and SCPs [67,90], biochemical characterization is still required to ascertain whether *GS5* acts as a peptidase or as an acyltransferase. Another clique, which extends only over *L. perrieri, Oryza sativa*, *Set. italica* and *So. bicolor* contains instead the gene *Os10g01134*, a putative flavonoid-specific AT [90], which is the closest relative to *AsSCPL1* (an authentic SCPL-AT isolated from *Avena sativa*, responsible for the decoration of avenacin with both *N*-methyl anthraniloyl and benzoyl moieties [37]). A further case of SCP/SCPL cliques limited to specific families are those of the Brassicaceae (figure 2*b*), among which two cliques derive from SCP/SCPL clade I (OG0000185). The Arabidopsis gene *At5g09640* (*sng2*, *sinapoylglucose accumulator2*) is a member of one of these cliques along with other genes from *Brassica napus*, *Brassica oleracea*, *Brassica rapa*, *Camelina sativa*, *Capsella rubella*, *Lepidium meyenii* and *Thellungiella parvula. sng2* has been characterized as a sinapoylglucose:cholinesinapoyltransferase (SCT), an AT involved in the synthesis of the seed-specific ester synapoylcholine [56,91]. This sinapate ester is only found in Brassicaceae [92], and the strong positional/reciprocal conservation of the key gene for its synthesis suggests a coordinated transcriptional regulation in Brassicaceae. Further research is required to clarify the role of the second Brassicaceae clique. SCP/SCPL genes are also shown to form some cliques extended specifically across Fabaceae (figure 2*b*), a plant family known to accumulate hydrolysable tannins, galloylated flavan-3-ols and galloylated glycosides of flavonols [28,93–96]. These metabolites, in other species, have been shown to be typical products of SCPL acyltransferases [58,97]. One of these Fabaceae-specific clique derives from clade I (OG0000185; see the electronic supplementary material, table S3), which contains, among other members, all authentic SCPL acyltransferases characterized so far. All members in this Fabaceae-specific clique from clade I contain the GDSYS motif (electronic supplementary material, figure S3), which so far has been diagnostic for all characterized SCPL-ATs grouped in clade IA [67].

Compared with BAHD, there are fewer SCP/SCPL cliques containing genes or gene regions spanning a wider range of species. Of these, one clique derives from clade I (OG0000185) contains the members of the flavonol-phenylacyltransferase (FPT) cluster characterized in Arabidopsis [52]. The clique contains 35 gene regions and spans 30 species. Its members include genes from Amborella, Monocots (*P. equestris*, *Brachypodium*, rice) and from several Eudicots (both Asterids and Rosids) and is positionally conserved, with the number of paralogues ranging from four (*T. parvula*) to 12 (Amborella). Although the clique, as a network object, underestimates the full range of species showing synteny (given that there could be other marginal nodes, from additional species, connecting to the genes in this clique), conservation of the *FPT* cluster is thus more extended with respect to what has been previously reported [52], as presence of *FPT* genes can be traced back to Amborella with the same genomic organization also found in the other Eudicots.

Acylated flavonoids, of a type similar to those produced by the SCPL *FPT* genes, can be also produced by BAHD genes, and we thus investigated the cliques containing *NtMAT1*, a BAHD flavonoid malonyltransferase initially isolated and characterized in *N. tabacum* [38]. Particularly interesting here is that also *NtMAT1*—in addition to the SPCL *FPT* genes—belongs to a cluster of BAHD genes arranged in tandem. This BAHD cluster, which in *N. tabacum* is composed by six genes, can be also found, with a strong reciprocal positional conservation, across 18 other Angiosperm species, from Monocots to Eudicots, with a number of genes ranging from two (*M. esculenta*) to sixteen (*M. truncatula*). Thus, the cliques containing the members involved in the acylation of flavonoids, from both BAHD and SCPL, are extended across several Angiosperms. It thus seems that the biochemical convergence of BAHD and SCPL-AT enzymes toward the production of acylated flavonoids was also accompanied by a positional conservation extended over very similar species ranges.

To recapitulate, in taking a global overview of the clique distribution composition of BAHD and SCP/SCPL genes, some apparent commonalities emerge: both families possess large cliques which extend over a relatively large range of species, although it is rare that fully reciprocal synteny extends over all Angiosperms. This is a consequence of the multitude of whole-genome and small-scale duplications, introgressions, horizontal gene transfers and events of gene loss or conversion which have resulted in the large diversity of Angiosperm genome size and structure [78,98]. An increasing number of plant genome studies have in fact shown how frequent have been recent, plant lineage-specific WGDs with respect to the more rare ancient paleoploidy events at the base of Spermatophytes [12,99]. It is more common instead to recover cliques, for both BAHD and SCP/SCPL genes, which are limited to either Monocots or Eudicots (but not both). Another feature of the synteny patterns common to the two gene families is the recovery of cliques which are instead limited to specific families: Poaceae, Brassicaceae and Fabaceae—the latter being evident in SCP/SCPL genes only. While the extended synteny represented by those few large cliques can be ascribed to the ε duplication (around 300 Ma, predating the split of Monocots from Eudicots), these family-specific syntenies probably emerged following the more recent WGD events at the base of each specific family, i.e. the τ/σ WGD event for Monocots/Poaceae, the At-alpha for Brassicaceae and the WGD event at the base of Faboideae [78,99], and were maintained in connection to the specific metabolites these species developed, as was the case for example for the sinapate esters of Brassicaceae [28,92,100] or the aromatic phenolamides of the Poaceae [101,102].

Detecting global differential patterns in the conservation of synteny between BAHD and SCPL-ATs remains a complex issue. If, on the one hand, all BAHD act as genuine acyltransferases, on the other SCPL emerged from SCPs (peptide hydrolases) acquiring, at some point during their evolution, a transacylation capacity. At the sequence level, there are no clear marks distinguishing these two activities, except for the experimentally characterized SCPL-ATs from clade Ia [103], which are grouped in a large OG containing also several SCPs (OG0000185, table 1). SCP/SCPL clade I (containing SCPL-ATs) and II (SCPs) have also very similar times of emergence and undergo across evolution to almost the same CNV pattern (few members in Heterokontophyta and algae, with an expansion in Gymnosperms and Monocots, figure 1*c*). The similar dynamic changes affecting these OGs are thus not suggestive of a CNV pattern which may discriminate SCPL-ATs from SCPs. If we look specifically at the few SCP/SCPL cliques derived from clade I (figure 2*b*), those that contain putative SCPL-ATs (i.e. having the pentapeptide GDSYS, where the central S is the catalytic Ser, [67]), have either a family-wide species range (like the Brassicaceae-specific clique containing *At5g09640*, *sng2*), or span over a larger range, from Monocots to Rosids, in a way similar to what has been observed for BAHD cliques (figure 2*a*).

### (d) Topological characteristics of the BAHD and serine carboxypeptidase/serine carboxypeptidase-like network components

To look deeper into the synteny structure of BAHD and SCP/SCPL genes, we decomposed the core synteny network into its components defined by the OGs to calculate, separately for the two gene families, a series of topological parameters. We aimed, in this case, to uncover some differences between BAHD and SCP/SCPL gene families, either at the global gene family level, or at the level of the single network components. Looking at the topological parameters of the components also allowed us to extend the analysis of synteny from the complete subgraphs—represented by the cliques (which represent a form of ‘cleaner’ synteny construct)—to the more complex network objects represented by their components. Components, contrary to cliques, are not complete subgraphs, and thus contain the additional synteny relationships brought by marginal nodes. This occurs since on transforming synteny into a quantitative measure, there might be some very weak syntenic relationships that do not form an edge. Network topology was used already in the field of synteny to detect major differences between taxa or to clarify incongruent phylogenetic relationships [6,98].

**Figure 3.**
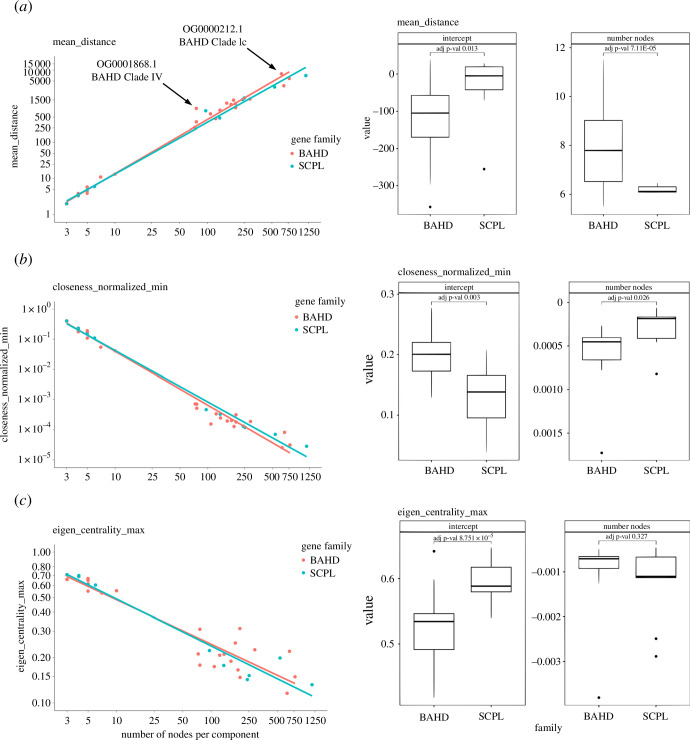
Topological parameters of BAHD and SCP/SCPL synteny components. In all scatter plots (left), the *y*-axis represents the value of the topological parameter and the *x*-axis represents the number of nodes in each component. Each component is represented as a dot (orange for BAHD and turquoise for SCP/SCPL). The box plots on the right show the distribution of the bootstrapped values for the regression coefficients (intercept and number of nodes). (*a*) *x–y* scatter plot and box plot for the mean distance (average path length). The regression lines through BAHD and SCP/SCPL components differ both in the slope and in the intercept. (*b*) *x*–*y* scatter plot and box plot for the normalized closeness (minimum value). Also here, the regression lines differ in the slope and intercept; (*c*) *x*–*y* scatter plot and box plot for the eigenvalue centrality (maximum value). In this case, the regression lines through BAHD and SCP/SCPL synteny components are significantly different only in the intercept.

For each component, we thus plotted the value of the specific topological parameter against the number of nodes in a *x–y* scatter plot (figure 3). In some cases, the regression lines fitting the BAHD and the SCP/SCPL components showed significant differences either in their slopes and/or intercepts, an indication that some specific topological parameters efficiently captured—on the global scale—a different structure of the synteny networks between these two gene families. Indeed, the slopes of regression for some centrality measures [104] showed significant differences for BAHD and SCP/SCPL genes (table 3), indicating a different distribution (i.e. range) of these values across the components of the two families. Centrality measures are frequently used to characterize networks. They have been used to assign specific values to the nodes on the basis of their connection topology. In regulatory and metabolic networks, be they composed by proteins or metabolites, highly important, indispensable nodes for organisms’ fitness often score high on several centrality measures [105]. In synteny networks, rather than indicating essentiality, the value of the topological parameters (either for single nodes or entire components) can be interpreted in terms of the extent of synteny conservation, expansion or contraction of gene families, and the capacity of incipient paralogues to be retained after genome duplications and other forms of genomic rearrangements [3].

**Table 3.** Topological parameters showing significant differences in the coefficients (slopes and/or intercepts) of the regression lines across BAHD and SCP/SCPL synteny components. (Values in bold denote significant adjusted *p*-values (<0.05).)

	slope (number of nodes)			Intercept		
topological parameter	*t* statistic	d.f.	adj. *p*‐value	*t* statistic	d.f.	adj. *p*‐value
mean distance (=average path length)	5.500	30.333	**7.112 × 10^−5^**	−3.156	23.440	**0.013**
betweenness (normalized, max)	2.602	18.165	**0.0438**	−8.375	19.378	**5.666× 10^−7^**
closeness (normalized, min)	−2.878	21.739	**0.026**	4.216	13.804	**0.003**
closeness (normalized, max)	−1.552	14.339	0.232	2.889	17.737	**0.026**
constraint (median)	−2.227	26.809	0.067	3.878	19.215	**0.003**
diameter	5.233	31.592	**1.019 × 10^−4^**	−6.113	38.876	**2.377× 10^−6^**
degree (normalized, min)	−2.877	12.318	**0.038**	10.484	28.128	**6.212 × 10^−10^**
degree (normalized, median)	−3.640	29.239	**0.005**	8.977	19.094	**2.715 × 10^−7^**
degree (normalized, max)	−4.525	35.471	**4.230 × 10^−4^**	5.311	15.281	**4.000 × 10^−4^**
eccentricity (min)	2.829	12.213	**0.039**	−1.667	14.995	0.174
eccentricity (max)	3.293	30.336	**0.009**	−0.889	15.398	0.473
edge betweenness (max)	−11.196	22.906	**3.567 × 10^−9^**	5.620	10.493	**8.034 × 10^−4^**
eigen centrality (min)	−4.945	38.942	**1.17× 10^−4^**	10.533	33.175	**1.589 × 10^−10^**
eigen centrality (median)	−3.422	24.195	**0.009**	1.708	14.116	0.171
eigen centrality (max)	1.218	14.232	0.327	−5.139	30.028	**8.751 × 10^−5^**
eigen centrality value	1.969	17.697	0.110	−2.561	12.289	**0.048**
neighborhood size (max)	−0.241	10.358	0.858	−2.634	10.415	**0.048**
strength (min)	−2.271	27.952	0.064	9.513	25.077	**1.097 × 10^−8^**
strength (median)	−4.569	17.052	**1.51 × 10^−3^**	1.875	12.928	0.148
distances (median)	7.986	27.016	**2.704 × 10^−7^**	−1.519	11.936	0.218
distances (maximum)	3.585	32.388	**4.729 × 10^−3^**	−4.071	38.961	**8.064 × 10^−10^**

An important difference between BAHD and SCP/SCPL components is in the regression coefficients of the mean distance (i.e. average path length, table 3; figure 3*a*). This parameter measures the average of all shortest paths between all pairs of nodes in each component. Generally, BAHD components have higher average path lengths with respect to SCP/SCPL components, indicating that synteny across BAHD components is more extended owing—presumably—to the inclusion of several marginal BAHD nodes on the periphery of the multispecies synteny blocks. This is particularly evident in OG0000212 (BAHD clade Ic) and OG0001868 (clade IV, which contains most of the BAHD *N*-acyltransferases) in comparison to those SCP/SCPL components which have a similar number of nodes. The larger extension of synteny of BAHD is also reflected in the network diameter, generally higher for BAHD components with respect to SCP/SCPL, with significant differences both in the slope and the intercept of the regression lines (table 3). Diameter is a parameter related to mean distance, and represents the maximum value of all shortest path lengths in each component. The higher diameter values of the BAHD components can be interpreted in terms of the wider phylogenetic extension of BAHD synteny, suggesting a higher resilience of BAHD, with respect to SCP/SCPL, in maintaining their genomic context despite the events causing dispersion of synteny.

Closeness centrality of a node, for example, is an indication of how close the node is to all other nodes in a specific component; it is a parameter specifically measuring the number of steps required to reach every other node from a given vertex. The distribution of these values (minimum and maximum closeness centrality score within each component) is different between the two gene families: BAHD components have generally lower minimum and maximum closeness values with respect to SCP/SCPL, indicating that their synteny embraces several marginal genes or gene regions which are located on the periphery of the component, and which have generally lower degrees (so few synteny relationships). SCP/SCPL components have instead more compact synteny structure, with genes generally closer to each other and less dispersed. This is also confirmed by eigenvector centralities, a measure scoring high those nodes which are connected to many other highly connected nodes (electronic supplementary material, table S5, contains the full data regarding the topological parameters for all BAHD and SCP/SCPL components).

Thus, the larger sequence (and phylogenetic) diversification BAHD genes accumulated during evolution was accompanied by a sparser synteny structure, extended over a wider range of species and embracing peripheral nodes. This is an indication of a stronger conservation of genomic context in this gene family, conservation which has been more strongly maintained in the BAHD than in the family of SCP/SCPL genes. This is suggestive of a general phenomenon indicating that the processes at the basis of gene and genome divergence (e.g. polyploidization and subsequent gene loss, neo and subfunctionalization of gene duplicates, hybridization, etc.) had different impacts on the two gene families as reflected by the changes observed in the composition, extent and topology of the synteny network components [98].

## 3. Conclusions

The study of the evolution of metabolism in plants is a challenging field. The extant chemodiversity is so vast that our current analytical capabilities can capture only a fraction of it, with the result that our knowledge of plant metabolism is limited to those cases in which individual enzymes are experimentally characterized to measure their binding and kinetic properties. As discussed both in the introductory comments [106], in our analyses above, and in subsequent articles in this theme issue [106], the complexity of plant metabolism is also and in large part owing to the inherent complexity of the genes encoding the decorative enzymes which chemically modify core structures of the major classes of plant specialized metabolites. Many of these enzymes are catalytically promiscuous and thereby operate at multiple points within the complex metabolic networks that constitute plant specialized metabolism [107,108]. In a recent study, we developed a computational pipeline to evaluate phylogenetic and synteny relationships across whole-genome sequences of the green lineage with the aim to understanding the evolution of metabolic capabilities at the level of individual gene (enzyme) families [5]. Our previous study focused on the type III polyketide synthase superfamily (PKS), whose ancestral members probably evolved from β-ketoacyl acyl carrier protein synthases of fatty acid metabolism. PKSs are highly important enzymes as they provide precursors for the synthesis of flavonoids and sporopollenin. In combining the analysis of genomic location with changes in gene sequences, the study revealed that the two major clades of the type III polyketide synthases, i.e., chalcone synthase and less adhesive pollen 5 and 6 (LAP5/6), evolved early by segmental duplication prior to the divergence of Bryophytes and Tracheophytes, with further diversification being governed by WGDs and triplications. We thus exploited the computational resources developed for this study to analyse the BAHD and SCP/SCPL gene families, whose members encode enzymes which have been found to be responsible for a large range of the acylation reactions involving secondary metabolites. Our analysis here revealed that SCP/SCPL genes were more deeply evolutionarily rooted than BAHD genes, which expanded massively on the transition to land and also later, in parallel with the development of the vascular system. The two gene families additionally displayed quite distinct patterns of CNV across phylogenies as well as differences in cross-phylogenic syntenic network components, both between themselves and in comparison with the polyketide synthase superfamily. Our analyses thus extends on previous demonstrations of the possibilities afforded by modern phylogenomic (syntenic) network analyses, which have been afforded at different scales, from biological kingdoms to single gene families [5,6,18], but on the other also highlights its current limitations, as demonstrated by the inability of phylogenetic methods to separate authentic SCPL acyl transferases from their ancestral forms, the serine carboxypeptidases. This fact notwithstanding, to our knowledge this is the most extensive analysis presented for these two gene families. As such, it provides considerable insight into the evolutionary trajectories of two important gene families shaping plant chemical diversity, as well as reinforcing the general value of the analysis of synteny as a crucial component of the toolbox for genome-based studies of the evolution of plant metabolism.

## 4. Methods

### (a) Collection of genomic and proteome data

The initial genome and proteome files used here were initially collected for the analyses reported in [5]. Briefly, genome and proteome files were downloaded from public repositories for all species listed in the electronic supplementary material, table S1, cleaned to remove the presence of minor splice variants and transposable elements, and checked for proteome completeness using BUSCO [109]. The final set thus included 126 high-quality genomes and proteomes, comprising Cyanobacteria (one species), Heterokontophyta (two species), Algae (10 species: two Rhodophyta, one Glaucophyta, five Chlorophyta and two Charophyta), non-vascular plants (two species), early Tracheophytes (three species), Gymnosperms (five species) and Angiosperms (103 species, of which one basal Angiosperm species—Amborella—plus 19 Monocots and 83 Eudicots).

### (b) Inference of orthologues and orthogroups

The cleaned FASTA proteome files were used as inputs for OrthoFinder v. 2.4.0 [31], with default settings (using diamond as protein aligner, dendroblast to build distance matrices and fastme for gene tree inference). The all-versus-all Blast results from OrthoFinder were also used to determine MCL groups according to [32].

### (c) Retrieval of BAHD and serine carboxypeptidase-like acyltransferase genes

Retrieval of BAHD and SCPL acyltransferases gene sequences was based on the recent genomic surveys and databases for these two families [22,29,30,35,36,77]. In essence, the identities of ‘bait’ genes (biochemically characterized BAHD and SCPL, listed in table 1) were used to retrieve their respective OGs from the OrthoFinder output. Clade numbers were then assigned to the various BAHD/SCPL OGs, based on the presence of specific acyltransferase genes in a specific OG and on previous phylogenetic reports for these families [22,29,34]. On the basis of the OrthoFinder output, the original BAHD clade I, as defined by [34], was split into clade Ia (OG0000346) and clade Ib (OG0000212), clade III was split into clade IIIa (OG0000119) and IIIb (OG0001133), and genes initially belonging to clade V were split into three OGs (OG0000161, OG0000365 and OG0001959). Similarly, the data from OrthoFinder refined the initial SCP/SCPL classification, based on [29], yielding six OGs. The number of BAHD and SCP/SCPL genes belonging to each OG are reported in table 1 and the electronic supplementary material, table S2. For the analysis of CNV, the gene count from each species/OG was used as input to draw heatmaps.

### (d) Identification of syntenic regions

Syntenic regions were identified with the algorithms and procedures described in [5]. For synteny detection we used the synteny detection algorithms i-ADHoRe and MCScanX. Starting from a gene homology matrix, i-ADHoRe uses an iterative algorithm to detect syntenic regions followed by statistical validation of the clusters in the gene homology matrix. MCScanX uses dynamic programming to find chains of collinear gene pairs. Further details are provided in [110,111]. Briefly, we first assembled, from the .gff files, a new file for each chromosome/species containing information about the genes and their orientation (±). These files were used as inputs for i-ADHoRe (v3.0.01) to detect collinear regions between two genomes. In an alternative approach, the outputs from OrthoFinder and MCL clustering were also used as inputs in MCScanX to detect syntenic regions [111]. Synteny relationships were thus assigned only if a specific gene pair was considered collinear following both i-ADHoRe and MCScanX.

### (e) Construction of phylosynteny networks

Assembly of the global phylosynteny networks for BAHD and SCPL-containing genomic regions was achieved using a custom script described in [112].

based on the approach initially developed by [3,6]. The script takes as input the result files from the i-ADHoRE and MCScanX runs, and assigns tandem genes and syntenic relationships. As a result, a global network was created incorporating information on synteny from OrthoFinder+i-ADHoRe, MCL+i-ADHoRe, OrthoFinder+MCScanX and MCL+MCScanX results.

In short, the resulting phylosynteny network is a construct made of nodes (or vertices, each representing an individual gene or a genomic region containing a tandem array of genes) and edges (lines), which connect the syntenic genes or the gene regions. Edge weights were assigned—in steps of 0.25, on the basis of the number of times synteny was detected applying the algorithms specified above (e.g. an edge weight of 1 was assigned to those synteny relationships detected from all four approaches: OrthoFinder+i-ADHoRe, MCL+i-ADHoRe, OrthoFinder+MCScanX and MCL+MCScanX results).

The global network was checked for its quality, e.g. by showing the distribution of the type of links between nodes or by displaying the scaffold length of singleton nodes or of multiple nodes within the other network components. All subsequent analyses were performed on the six largest components of the initial network (core network). All network analyses were conducted in the statistical programming language R (v4.4.0) with the dplyr (v1.1.4), ggplot2 (v3.4.4), ggpubr (v0.6.0), igraph (v2.0.1) and the scales (v1.3.0) packages.

### (f) Clique determination

A clique in a network is an induced subgraph that is complete, i.e., where every node is directly connected to every other node in the subgraph. Starting with the core network, all edges with weights greater than 0 were set to 1. For each of the six components of the network, we selected the largest clique, the largest induced subgraph that is complete, from the subnetwork and removed the members of the clique from the subnetwork. In case of multiple largest cliques at a given step, we randomly selected one of the largest cliques and removed the members of the clique from the subnetwork. To find a local optimum, this iterative (random) selection was repeated 100 times for each component and the result with the lowest total number of cliques and with the highest median number of membership per clique was selected. The corresponding analysis can be found via [113].

### (g) Community detection

A network community is a group of nodes (subset) showing higher density of connections within the subset with respect to the other network nodes which are not part of the subset. On the core network, the following community detection algorithms, using default parameters unless indicated otherwise, were applied taking into consideration the edge weights: via greedy optimization of modularity(cluster_fast_greedy), via short random walks (cluster_walktrap, steps = 15), via the leading eigenvector of the community matrix (cluster_leading_eigen, steps = 15), via propagating labels (cluster_label_prop), via multi-level optimization of modularity (cluster_louvain) and via Infomap community finding (cluster_infomap, nb.trials = 100). The corresponding analysis can be found in [113].

### (h) Calculation of topology information

The core network consisting of OrthoFinder and MCL information was decomposed into components of the same OG (minimum number of nodes = 3). On these components, consisting either of BAHD- or SCP/SCPL-containing genomic regions, topology parameters were calculated as outlined in the electronic supplementary material, Script. Linear models were separately created from the BAHD and SCP/SCPL data points using the number of nodes per component as the independent variable and the topology parameter value as the dependent variable. To determine differences between the linear models from BAHD and SCPL data points, these data points were resampled via bootstrapping to build multiple linear models for BAHD and SCPL (number of resampling: 11 and 30 for SCP/SCPL and BAHD data points, respectively). Differences between linear model coefficients (intercept, slope) were tested using *t*-tests (*α* < 0.05, false discovery rate adjustment via Benjamini–Hochberg method). The corresponding analysis can be found in [113].

## Data Availability

Links to Zenodo are provided in §4 of this work [112,113]. Supplementary material is available online [114].
